# The loop structure and the RNA helicase p72/DDX17 influence the processing efficiency of the mice miR-132

**DOI:** 10.1038/srep22848

**Published:** 2016-03-07

**Authors:** Judit Remenyi, Sarah Bajan, Frances V. Fuller-Pace, J. Simon C. Arthur, Gyorgy Hutvagner

**Affiliations:** 1 Division of Cancer Research, Jacqui Wood Cancer Centre, University of Dundee, Ninewells Hospital and Medical School, Dundee, UK; 2 Division of Cell Signaling and Immunology, College of Life Sciences, Wellcome Trust Building, University of Dundee, Dundee, UK; 3 Faculty of Engineering and Information Technology, Centre for Health Technologies, University of Technology Sydney, NSW 2007, Australia.

## Abstract

miRNAs are small RNAs that are key regulators of gene expression in eukaryotic organisms. The processing of miRNAs is regulated by structural characteristics of the RNA and is also tightly controlled by auxiliary protein factors. Among them, RNA binding proteins play crucial roles to facilitate or inhibit miRNA maturation and can be controlled in a cell, tissue and species-specific manners or in response to environmental stimuli. In this study we dissect the molecular mechanism that promotes the overexpression of miR-132 in mice over its related, co-transcribed and co-regulated miRNA, miR-212. We have shown that the loop structure of miR-132 is a key determinant for its efficient processing in cells. We have also identified a range of RNA binding proteins that recognize the loop of miR-132 and influence both miR-132 and miR-212 processing. The DEAD box helicase p72/DDX17 was identified as a factor that facilitates the specific processing of miR-132.

The majority of metazoan miRNAs are encoded in the introns of PolII transcribed RNAs. The first step in the processing of miRNAs takes place in the nucleus; during this step the hairpin-like structured primary miRNAs (pri-miRNA) are recognized and cleaved by the Microprocessor complex, which contains Drosha, an RNAseIII enzyme and the RNA binding protein DGCR8^1^,^2^,^3^. The released precursor miRNA (pre-miRNA) is then exported to the cytoplasm by Exportin 5^4^. The pre-miRNA is further processed into a mature miRNA by Dicer, another RNAseIII enzyme, and one strand of the miRNA is loaded onto one of the Argonaute proteins, forming the minimal miRNA induced silencing complex (miRISC)^5^,^6^,^7^.

An increasing amount of evidence shows that the steady state level of miRNAs are post-transcriptionally regulated at diverse steps of miRNA maturation^8^. Many of the identified proteins that influence miRNA processing alter the activity of the Microprocessor and regulate the pri-miRNA to pre-miRNA conversion of a subset of miRNAs. For example, it has been found that SMAD^9^, p53^10^, hnRNPA1^11^ and KSRP^12^ facilitate the processing of certain pri-miRNAs. On the other hand, Lin-28 can inhibit the action of the Microprocessor^13^. miRNA maturation may also be regulated at the pre-miRNA processing step. For instance, Lin-28^14^ and MCPIP1^15^ can initiate the degradation of the bound miRNA precursors while KSRP is necessary for the efficient pre-miRNA processing for a subset of miRNAs^12^.

miR-132 and miR-212 are two related miRNAs that have been functionally linked to brain development and multiple neuronal processes such as circadian rhythm, addiction, ocular dominance, neuronal plasticity and long term potentiation^16^,^17^,^18^,^19^,^20^,^21^,^22^,^23^,^24^,^25^. miR-132 has also been implicated in immune response to viral infection^26^ and an increasing number of studies suggest that it has a role in cancers^27^,^28^,^29^.

One interesting feature of the mouse miR-132/212 cluster is that it leads to significantly higher levels of mature miR-132 than miR-212 despite the fact that they are co-transcribed and co-regulated^30^,^31^.

Here we present evidence that the mechanism for the uneven processing of the co-regulated miRNAs, miR-132 and miR-212 in mice is determined by the structure of the miR-132 loop. We also identified multiple RNA binding proteins that specifically bind the loop sequence of miR-132 and influence miRNA processing. One of these proteins is the DEAD box RNA helicase p72/DDX17 which, together with the highly related p68/DDX5 protein, is associated with the Drosha complex and is required for processing of specific subsets of miRNAs^3^,^9^. Our data show that p72/DDX17 specifically interacts with the miR-132 loop sequence and influences the relative ratio of the mature mice miR-212/132 miRNAs.

## Results

### Uneven processing of the **miR**-212/132 cluster does not depend on the specific cellular context

**Figure 1:**
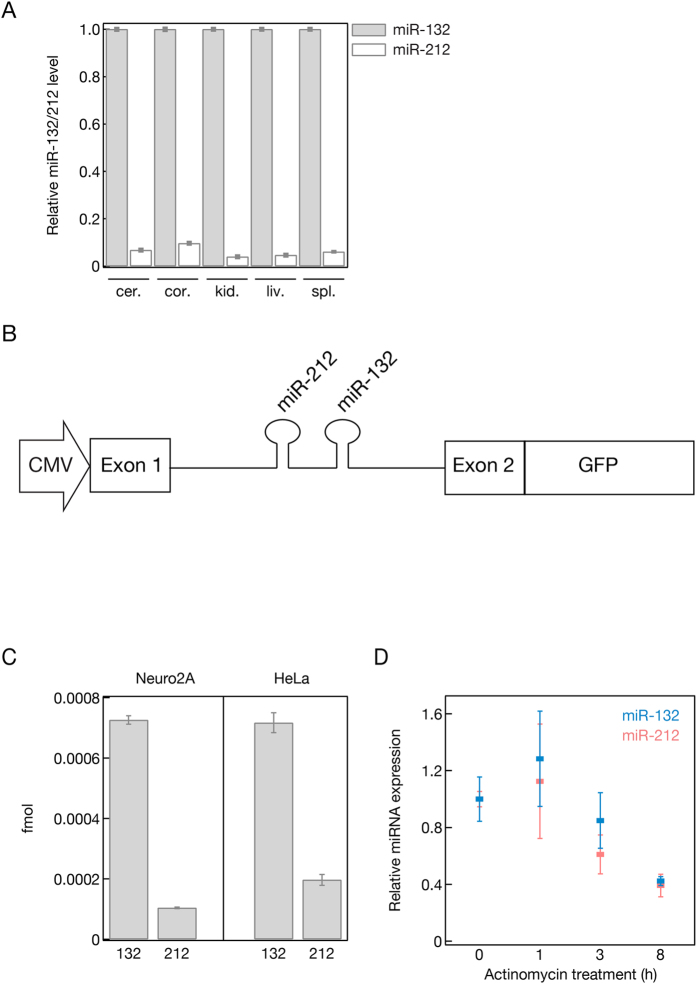
(**A**) mmu-miR-132 is more abundant in mice tissues compared to the co-expressed and co-regulated related mmu-miR-212. Relative mmu-miR-132 and mmu-mir-212 expression was measured by qPCR and compared in mice cerebellum (cer.), cortex (cor.), kidney (kid.), liver (liv.), spleen (spl.). (**B**) Schematic representation of the GFP reporter construct used to study miR-212/132 expression *in vitro*. (**C**) The unequal processing of mmu-miR-132 (132) and mmu-miR-212 (212) can be recapitulated *in vitro* in mice (Neuro2A) neuroblastoma and human (HeLa) cells using the reporter described in (**B**) The plasmid was transfected into both cell lines and the level of miR-132 and miR-212 were measured by qPCR. The absolute levels of the miRNAs were plotted. (**D**) The unequal processing of miR-132 and miR-212 is not the consequence of their different turnover rate. Hela cells were transfected with GFP reported plasmid (**B**) and PolII transcription was inhibited up to 8 hours by administering 1.0 μg/ml Actinomycin D. The level of miR-132 and miR-212 was measured with qPCR and compared to the level of DMSO treated cells before Actinomycin D addition.

We have previously reported that there is a significant difference between the steady state levels of mature miR-132 and miR-212 in primary cortical neurons isolated from mice, in spite of the fact that they are co-transcribed in the same intron of a non coding gene^30^. In order to test whether this is characteristic of this particular cell type or it is a general phenomenon we measured the relative miR-132/212 levels in several murine tissues (Fig. 1A). Our data show that miR-132 is significantly more abundant than miR-212 in each of the cells and tissues we examined, suggesting a general mechanism that favors the accumulation of miR-132 over miR-212.

### Uneven processing of the mouse miR-212/132 cluster can be recapitulated in cultured mammalian cells

To test whether this uneven processing can be recapitulated in cultured laboratory cell lines we generated a reporter plasmid (mmu-miR-212/132::GFP) that encodes the mouse pri-miRNA-212/132 bearing intron, flanked by its two endogenous exons. Additionally, the exon downstream from the pri-miR-212/132 containing intron was fused to GFP mRNA (Fig. 1B). Based on our detection of the mature miRNAs using Northern blotting and the GFP expression we concluded that the RNA transcribed from the reporter plasmid is properly spliced and both miRNAs were accurately processed in human HeLa and mouse neuroblastoma cells (Supplementary Figure 1A). We observed that the accumulation of miR-132 was significantly more prominent than the accumulation of miR-212 in both cell lines (Fig. 1C). This suggests that a conserved mechanism exists between mice and humans that favors the accumulation of mature miR-132 over miR-212 provided they are co-transcribed.

### The stability of the mature miR-212 and miR-132 is similar

The steady state miRNA level is not only influenced by auxiliary factors that regulate miRNA biosynthesis but could also reflect inherent differences between miRNA stability and turnover rate. To measure the stability of the two co-transcribed miRNAs we transfected HeLa cells with the miR-212/132::GFP reporter plasmid and inhibited RNA Pol II transcription using Actinomycin D. We collected RNA and protein samples at 1, 3, and 8 hours after Actinomycin D treatment and measured GFP levels by western blotting, and miR-212 and miR-132 levels using qPCR (Fig. 1D and Supplementary Figure 1C). For each time point we compared the protein and RNA levels to mock (DMSO) treated HeLa samples. When we measured the levels of miR-212 and miR-132 and compared these with the mock treated samples we could not detect any differences in their turnover rate (Fig. 1D). This result suggests that the uneven accumulation of these two miRNAs is not a result of different stability or turnover rate.

### The small RNA derived from the miR-132 loop sequence is not a miRNA

**Figure 2:**
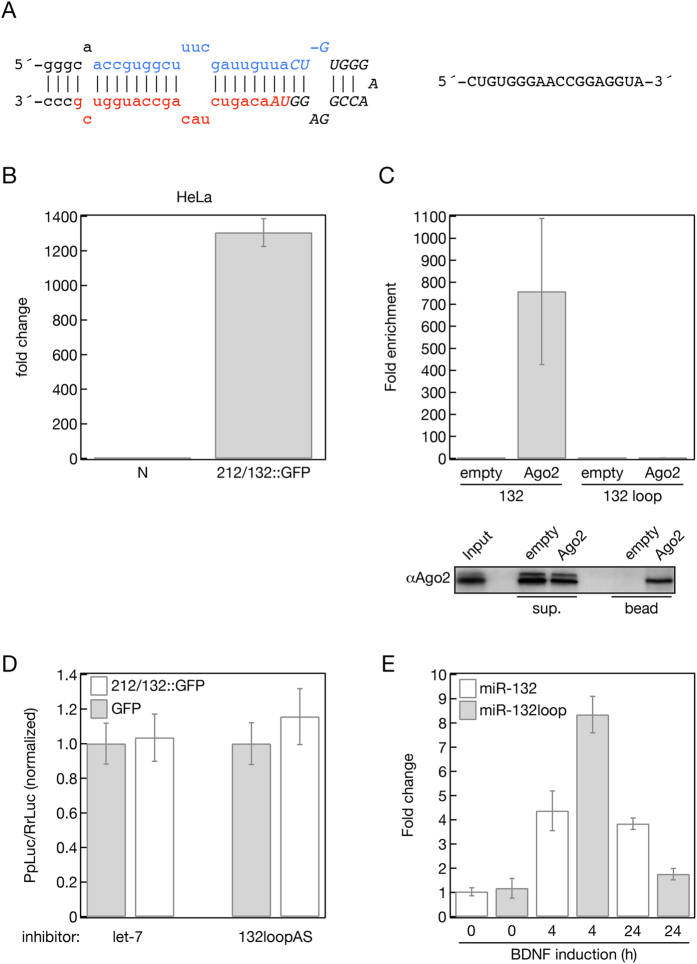
(**A**) The hairpin structure of the mice miR-132 pri-miRNA. The mature sense miRNA is blue and the *sequence is red. Capital letters with Italics highlight the accumulated sequence observed in deep sequencing experiments. This sequence is also shown right to the miR-132 hairpin structure. (**B**) The expression of the GFP reporter (212/132::GFP, Fig. 1B) also results in the accumulation of the 132 loop sequence in Hela cells measured by qPCR using loop specific primers and compared to non-transfected cell (N). (**C,D**) The miR-132 loop sequence is unlikely to be a miRNA. (**C**) Immunoprecipitation with human Ago2 antibody (Ago2) captured miR-132 (132) but failed to bind to the miR-132 loop sequence (132 loop). Hela cells were transfected with the 212/132::GFP (Fig. 1B) reporter plasmid followed by immuoprecipitation with Ago2 antibody. The efficiency of the immunoprecipitation was checked with western blot (bottom panel) using Ago2 antibody. Input represents of 0.5% of the total lysate while the Bead represents 2% of the captured Ago2. RNA was purified from the bound fractions, miR-132 and miR-132 loop sequence were quantified using qPCR and compared to the mock immunoprecipitation that was carried out without the use of Ago2 antibody (empty). (**D**) Inhibition of the miR-132 loop with specific inhibitor (132loopAS) did not increase the expression of a Luciferase reporter plasmid with multiple sites complementary to the miR-132 loop sequence in Hela cells that was co-transfected with the GFP reporter plasmid encoding the miR-212/132 cluster (212/132::GFP) Inhibitor for *let-7* and plasmid expressing GFP were used as a negative control. (**E**) Relative accumulation of the miR-132 mature and the miR-132 loop sequences in BDNF induced mouse cortical neurons. RNAs were purified from mouse cortical neurons after BDNF induction at the indicated times. The levels of miR-132 and miR132loop were quantified by qPCR and compared to their respective levels measured in the non-induced cells (0 time points).

Deep sequencing of brain-derived neurotrophic factor (BDNF) stimulated primary mouse cortical neuronal cultures revealed that the miR-212/132 miRNA cluster produces five small RNAs, miR-212, miR-212*, miR-132, miR-132* and a 18bp long small RNA derived from the loop of miR-132^30^ (Fig. 2A). The accumulation of the miR-132 loop small RNA could also be observed in human cells expressing the miR-212/132::GFP plasmid (Fig. 2B). Loop sequences for some other miRNAs have been detected by others in deep sequencing experiments, and in a few cases, it has been demonstrated that these RNAs could be *bona fide* miRNAs^32^,^33^. Therefore, we first tested whether the miR-132 loop RNA functions as a miRNA. A potentially functional miRNA has to be associated with a member of the Argonaute protein family and be able to mediate the cleavage of mRNAs that carry perfect complementary sequences to the miRNA. To test these properties, we transfected the miR-212/132::GFP construct into HeLa cells and 16 hours later we immunopurified (IP) endogenous Ago2, the main effector protein of the human RNA induced silencing complex (RISC). qPCR analysis of the Ago2 IP showed a strong enrichment of the mature miRNA-132 in the Ago2 bound fraction; however, no miR-132 loop was detected on the Ago2 beads (Fig. 2C). Next we tested whether the accumulated miR-132 loop sequence was capable of regulating a target RNA through RNAi in cells. We generated a Luciferase reporter plasmid that contained three perfect complementary target sites to the miR-132 loop sequence. The Luciferase plasmid was co-transfected with a GFP or miR-212/132::GFP expressing plasmid, together with a 2′-*O*-Methyl oligonucleotide targeting the mir-132 loop sequence or a 2′-*O*-Methyl oligonucleotide complementary to the human *let-7* as negative control. Figure 2D shows that neither the transfection of miR-212/132::GFP nor the inhibition of the miR-132 loop sequence affected the expression of the Luciferase reporter. Since the loop sequence was not enriched in the bound fraction of the Ago2 IP and the reporter designed to be targeted by the miR-132 loop was insensitive to this RNA we concluded that the miR-132 loop fragment is very unlikely to be itself a functional miRNA.

### The loop structure is important in regulating the processing efficiency of miR-132

**Figure 3:**
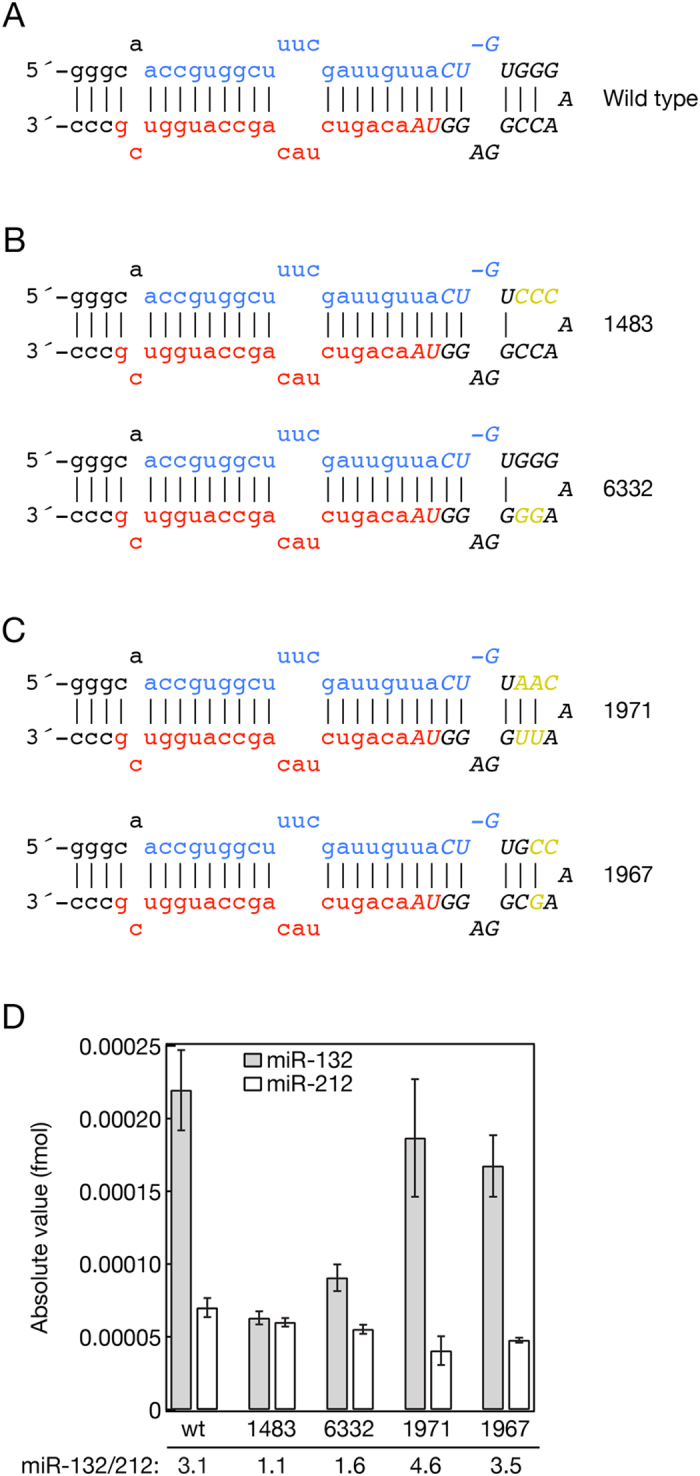
Mutating the loop structure in the GFP reporter plasmid (212/132::GFP, Fig. 1B) influences the efficiency mmu-miR-132 processing. (**A**) The hairpin structure of the mice miR-132 pri-miRNA. The mature sense miRNA is blue and the *sequence is red. Capital letters with Italics highlight the accumulated sequence observed in deep sequencing experiments. (**B**) Mutations (yellow sequences) were introduced to generate a more relaxed loop compared to the wild type miR-132 structure. (**C**) Reinstating the closed loop structure with sequences (yellow label) that are not part of the wild type miR-132 loop. (**D**) Absolute level of miR-132 and miR-212 produced by the transfected wild type and mutated 212/132::GFP reporter plasmids. miR-132 and miR-212 levels were quantified by qPCR and the absolute values were plotted. Numbers on the x-axis correspond to the numbers of respective mutations in (**A–C**). The relative miR-132/212 ratios are indicated below the graph.

An alternative explanation for the accumulation of the 18 nt long miR-132 loop sequence is that it may have a role in the efficient processing of miR-132. This is supported by the expression profile of the miR-132 loop sequence compared with miR-132 upon BDNF induction in mouse primary cortical neuronal cells (Fig. 2E). While miR-132 expression shows steady expression at 4 and 24 hours after BDNF induction the level of the miR-132 loop is much higher at the early time point. This would be consistent with the loop sequence providing a binding platform for auxiliary factors that promote miR-132 processing. This possibility is also supported by the discovery of an increasing number of regulatory proteins that influence miRNA processing by recognizing and binding to specific sequences and/or secondary structures of miRNA loops. To test this hypothesis, we generated a series of mutations in the loop of mmu-miR-132 in the construct described in Fig. 1B to investigate whether any of these modifications affect the efficiency of the processing of miR-132. We generated mutants in which we disrupted the relatively closed loop structure of the miR-132 (Fig. 3B: constructs 1483 and 6332) by mutagenizing the sequence that was recovered with deep sequencing (Fig. 3A). We also obtained constructs in which we reconstructed the loop structure using base pairings that are not part of the natural pri- and pre-mmu-miR-132 sequences (Fig. 3C: construct 1971 and 1967). Next, we transfected HeLa cells with the above-mentioned miR-212/132::GFP expressing plasmids and measured the concentration of both mature miR-132 and miR-212 using qPCR (Fig. 3D). The wild type construct again resulted in unequal processing of miR-132 and miR-212 by generating approximately 3 fold more miR-132 than miR-212. Interestingly, both mutants with the more relaxed loop structure significantly decreased the level of the mature miR-132 without affecting the quantity of miR-212, suggesting that the loop structure is necessary for efficient miR-132 processing. This is supported by the fact that the reconstitution of the closed loop reinstated the 3–5 fold differences between the levels of the mature miR-132 and miR-212 without significantly affecting the level of miR-212 (Fig. 3D).

Several studies showed that changes in the loop size or altering the distances between the loop stem junction and stem apical ssRNA junction of a pri-miRNA could affect Drosha cleavage efficiency and fidelity and could produce isomiR miRNAs with heterogenic ends^34^,^35^,^36^. In our mutants we did not change the stem length; however the incorporated mutations changed the size of the loop. In previous studies the size of loop determined the efficiency and fidelity of Drosha processing; however, most of the loop mutants had a deletion in the recently discovered apical loop sequence that is a key requirement for efficient Microprocessor activity^34^,^37^. None of our mutant constructs were mutated in the conserved apical UGU motif when we changed the structure of the miR-132 loop, but if the fidelity of the processing of the mutant miR-132 was compromised it would result in inefficient amplification by the Taqman PCR system, which is sensitive to the 3′ end of the miRNA sequence. To exclude the possibility that changing the loop structure affected the fidelity of miR-132 processing we repeated the experiment but this time we used Northern blotting which is inherently less sensitive to the heterogeneity of miRNAs, to detect and quantify miR-132 and miR-212 levels. This experiment produced very similar miR132/212 ratios to the qPCR result supporting our original observation that the loop structure influences miR-132 processing (Supplementary Figure 2).

### Identification of proteins that are associated with the miR-132 loop derived small RNA

After determination of the structural requirements for the proper processing of mmu-miR-132 we went on to isolate proteins that associate with the miR-132 loop that might regulate its processing. To purify proteins that bind to the loop sequence that was identified with deep-sequencing, we synthetized biotinylated 2′-*O*-Methyl oligonucleotides for affinity purifications. These included an oligo designed to mimic the sequenced miR-132 loop sequence and an oligo similar to the sequenced miR-132 loop but with the GGG motif replaced with CCC as a control. This modification in the miR-212/132::GFP reporter resulted in a decreased miR-132 level, suggesting that proteins that facilitate miR-132 processing may fail to bind to this sequence (Fig. 3B,D). Finally, an antisense oligo complementary to the sequenced miR-132 loop was generated as an additional negative control. We tested the miR-132 loop and CCC mutant oligos in a competition assay by cotransfecting them with the miR132/212::GFP reporter and we have found that the miR-132 loop 2′-*O*-Methyl oligo inhibited the accumulation of the mature miRNAs while the CCC mutant oligo had no effect on the miRNA processing (Supplementary Figure 3B.) Our data also shows that competing with both the miR-132 and miR-212 loop sequences results in the decrease of the level of the pri-miRNA, judging from the level of the surrogate GFP protein level. This may suggest that competing with proteins recognizing miRNA loop sequences on the pri-miRNAs prevent miRNA processing via the destabilization of the primary transcript. Therefore the loop oligo will also likely capture proteins that are involved in regulating pri-miRNA stability as well (Supplementary Figure 3A).

**Figure 4:**
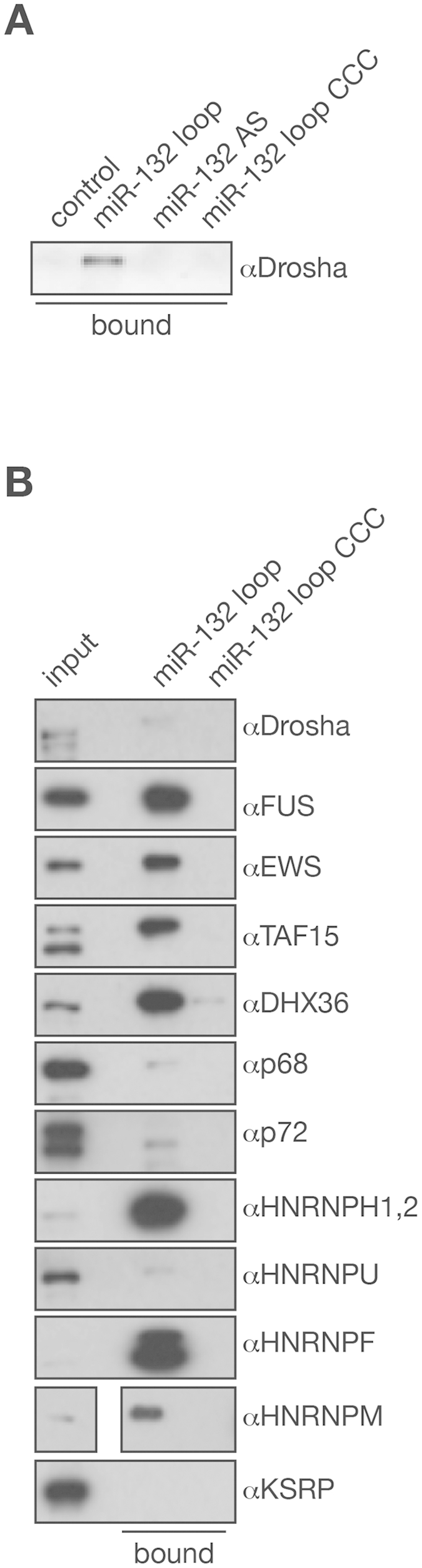
Validation of the binding specificity of the affinity purified miR-132 loop proteome. (**A**) Drosha specifically binds to the biotinylated miR-132 loop 2′-*O*-methyl oligo. (**B**) Affinity purification using biotinylated 2′-*O*-methyl oligos followed by Western blotting with proteins identified in proteomics experiment (Supplementary Figure 4). miR-132 loop: biotinylated 2′-*O*-methyl oligo mimicking the sequence derived from the miR-132 loop. miR-132 AS: biotinylated 2′-*O*-methyl oligo complementary to miR-132 loop. miR-132 loop CCC: biotinilated 2′-*O*-methyl oligo similar to miR-132 loop only three GGG sequence was replaced with CCC (see mutation in Fig. 3B clone: 1483). 2% of the total lysate was loaded as input and the beads represents 9% of the captured proteome.

The miR-132 loop was likely to be associated with the pri-miRNA processing machinery because the miR-132 loop mimic affinity purified with Drosha while neither the CCC mutant nor the antisense miR-132 loop control oligo were able to capture Drosha (Fig. 4A). In addition, neither Dicer nor Ago2 could be affinity purified by any of these oligos (data not shown), which supports the hypothesis that the relative accumulation of miR-132 is likely a consequence of a more efficient pri-miRNA processing. This was further supported by the fact that processing efficiencies of both pre-miR-132 and pre-miR-212 into their respective mature miRNAs were very similar (Supplementary Figure 1B). Next, we carried out a large-scale affinity purification with the two biotinylated control oligos and the biotinylated miR-132 loop sequence in duplicate. After capture and washing the bound fractions the purified proteomes were separated in 2D PAGE. We excised ten gel slices from the miR-132 loop affinity purification that showed specific accumulation of proteins and compared these with those from both negative controls (Supplementary Figure 4A). Mass spectrometry analysis of the samples showed that the miR-132 loop oligo predominantly bound to a wide range of RNA binding proteins, most notably members of the TET protein family (FUS, EWS and TAF15) and multiple HNRNP proteins (Supplementary Figure 4B). We also noticed that the captured proteome significantly overlaps with the proteome purified with Drosha and the *let-7* hairpin sequence in studies to identify proteins that regulate pri-*let-7* processing^38^,^39^. Next, we confirmed that a selected list of affinity purified proteins specifically bind to the miR-132 loop by repeating the pull down followed by Western blotting with the individual proteins. In this experiment we incorporated two additional proteins, p72/DDX17, a protein that is necessary for the processing of certain miRNAs and highly related to p68/DDX5 that was also present in the miR-132 loop purified proteome, and KSRP, which also has a well-described function in miRNA processing. These experiments showed that all the tested proteins with the exeption of KSRP were present in the bound fraction purified with the miR-132 loop oligo while they were absent from the beads obtained with the CCC mutant and the control oligo (Fig. 4B).

### Functional analysis of the miR-132 loop associated proteins

**Figure 5:**
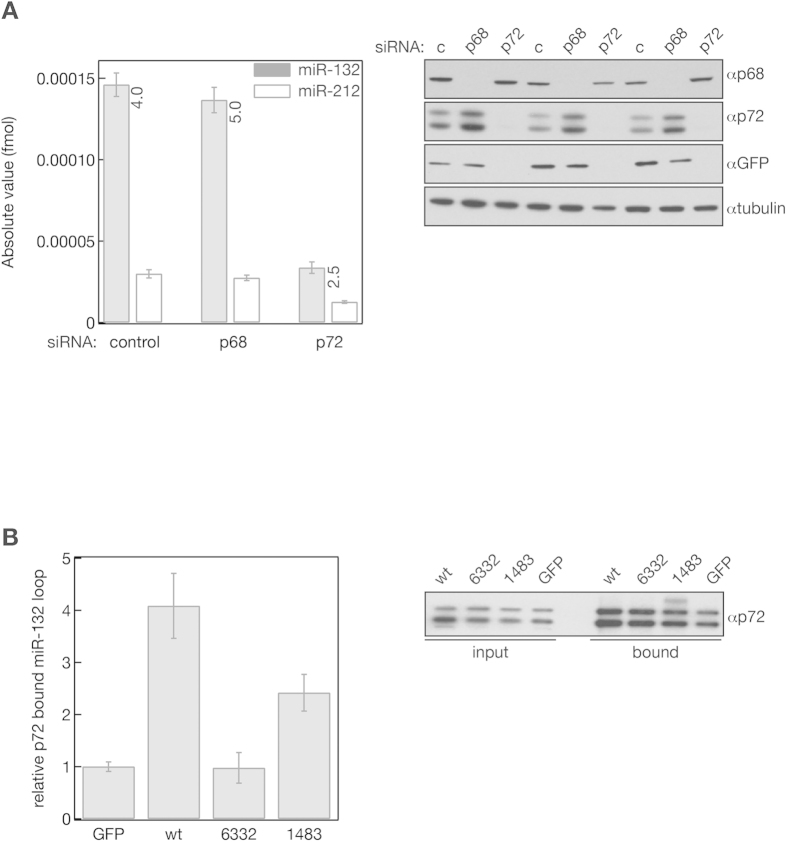
p72/DDX17 influence the stability of pri-miR-212/132 and the level of mature miR-132, miR-212 *in vitro*. (**A**) miR-212/132::GFP reporter was co-transfected with siRNAs against p68/DDX5 (p68) and p72/DDX17 (p72) into HeLa cells and the level of miR-132 and miR-212 was quantified using PCR. The ratios of mature miR-132/212 are indicated in the graph (left panel). The efficiency of the siRNA knockdowns was monitored with Western blotting using p68 and p72 antibodies and GFP antibody was used to measure the level of pri-miR-212/132::GFP (right panel). C and control: transfection carried out with non-targeting siRNA. (**B**) p72/DDX17 preferably binds to the wild type pri-miR-212/132::GFP reporter RNA. HeLa cells were transfected with GFP control plasmid (GFP), the miR-212/132::GFP reporter (wt) and two mutants with relaxed miR-132 loop sequences (Fig. 3B) followed by immunoprecipitation with p72 antibody. GFP antibody was used as a negative control for the IP. 2% of the total lysate was loaded as input and the bead represents the 2.5% of the pulled down proteins. The p72 and GFP bound miR-132 levels were quantified by qPCR (left panel). Relative levels of miR-132 immunopurified with p72 were compared to the miR-132 level associated with the control immunoprecipitation carried out with the GFP antibody. The efficiency and uniformity of the p72 IP was monitored with Western blotting (right panel).

To test if the affinity purified miR-132 loop associated proteins played roles in the processing of miR-132 we knocked down each of the proteins that bound selectively to the miR-132 loop sequence with siRNA in Hela cells that were then transfected with the miR-212/132::GFP reporter plasmid (Fig. 1B). 72 hrs after siRNA knockdown and 24 hours after transfection of the 212/132::GFP plasmid, the efficiency of the knock downs was measured by Western blotting (Fig. 5A right panel, Supplementary Figure 5A,B right panels). The level of miR-132 and miR-212 was quantified by qPCR and the absolute quantities of the two miRNAs were calculated and compared (Fig. 5A left panel, Supplementary Figure 5A,B left panels). GFP was used to measure the influence of the protein knock downs on RNA stability and/or splicing (Fig. 5A right panel, Supplementary Figure 5A,B right panels). We were looking for outcomes that are similar to the effect of miR-132 loop mutants that resulted in a decreased miR-132 processing without drastically changing the level of miR-212 (Fig. 3D). Some of the RNA binding proteins such as EWS, HNRNPM, HNRNPU and DHX36 showed an inhibitory effect on mmu-miR-212/132 processing and/or RNA maturation since knocking them down resulted in elevated miR-132, miR-212 and GFP expression (Supplementary Figure 5A). Downregulation of FUS, HNRNP2 and HNRNPF increased the GFP level without significantly affecting miR-132 and miR-212 levels suggesting that they may be involved in RNA maturation independently from miRNA processing. We also knocked down several co-purified RNA binding proteins simultaneously. Decreasing the expression of FUS and EWS increased the expression of GFP and both miRNAs (Supplementary Figure 5A). HNRNPH has two isoforms and they can form heterodimers with HNRNPF^40^ therefore we knocked down both isoforms simultaneously and also generated a triple knock down of HNRNPH1, 2 and HNRNPF. HNRNPH2 and HNRNPF negatively regulated mRNA maturation without affecting miRNA processing while eliminating both isoforms of HNRNPH together with HNRNPF largely decreased the expression of both miRNAs and GFP (Supplementary Figure 5B). p72 was the only protein that influenced the level of miR-132 more than the processing of miR-212 since it’s downregulation significantly decreased the ratio of the mature miRNAs generated from miR-212/132::GFP (Fig. 5A). However, impairing p72 expression also had a detrimental effect on GFP expression suggesting that p72 is not only required for proper miR-212/132 processing but is also involved in the maturation of the GFP mRNA. To further confirm that p72 specifically recognizes the miR-132 loop, we immunoprecipitated endogenous p72 from HeLa cells that were transfected with wild type mmu-miR-212/132::GFP and the two loop mutants (6332 and 1483 in Fig. 3) that showed impaired miR-132 processing. RNA was isolated from the bound fractions and the miR-132 loop sequence RNA was quantified by qPCR. The miR-132 loop enrichment was calculated by comparing the results to a bound fraction of a p72 immunoprecipitate carried out from control GFP transfected Hela cells (Fig. 5B). Our data shows that p72 preferably binds to the wild type miR-132 loop supporting the notion that it may be involved in miR-132 processing by recognizing the loop sequence we identified by deep sequencing.

## Discussion

In this study, we dissect the molecular mechanism responsible for the asymmetric production of the co-transcribed miRNAs, miR-212 and miR-132 in mice. We have shown that the relatively closed loop structure of miR-132 is a key determinant for the favorable processing of miR-132 over miR-212. To further understand this we sought to identify auxiliary factor(s) that promote miR-132 processing. This experiments included both affinity purification with the miR-132 loop sequence and a candidate approach involving testing auxiliary factors with known function in miRNA processing. This identified several RNA binding proteins that specifically bind to the miR-132 loop. Among these, only p72 showed preference for facilitating miR-132 maturation, however; knocking down many of the affinity-purified proteins altered the level of the pri-miR-212/132 and/or the abundance of both miRNAs. This is not surprising since the competition with the miRNA loop sequences showed that the loops are important in the stability of the pri-miRNA at least in our reporter system.

miRNA biogenesis starts with the recognition and processing of miRNA containing hairpin loops in the pri-miRNAs by the Microprocessor complex^2^,^3^. The Microprocessor also recognizes other hairpin structured RNAs but its primary targets are miRNAs^41^,^42^. This relative specificity and efficiency of the Microprocessor in the processing of miRNAs is partially determined by primary and secondary structure elements that seem to be characteristic to the majority of mammalian pri-miRNAs^37^,^43^,^44^,^45^. During preparation of this manuscript, a UGU sequence at the apical junction of the mammalian pri-miRNAs was suggested to promote pri-miRNA processing^37^. Very recent biochemical data confirmed that DGCR8 preferably recognizes the apical UGU sequence which facilitate productive pri-miRNA cleavage^45^. After closer examination of the human and murine miR-132/212 sequences it became clear that the apical UGU sequence is intact in miR-132 but it is missing from miR-212. This could partially account for the uneven processing of the two related miRNAs. However, when we generated mutants in the loop of mmu-pri-miR-132 the UGU sequence was unaltered in the mutants that showed impaired processing. This suggests that other factors in addition to the UGU sequence, exist that determine the efficiency pri-miR-132 processing.

An increasing amount of evidence indicates that the miRNA loops provide binding platforms for auxiliary proteins that regulate accessibility and processing, not only pri-miRNAs but also pre-miRNAs. hnRNPA1 was the first protein identified to promote the processing of an individual miRNA among the co-transcribed miR-17-92 miRNA cluster by binding to a conserved sequence motif in the loop of miR-18a and generated a more relaxed structure necessary for efficient processing^46^. A further study also suggested that strong conservation of the loop sequences in miRNA families is very likely a sign to accommodate such auxiliary partners, mainly proteins from the hnRNP RNA binding protein families^47^. Our findings show similarities to the regulation of the processing of the human miR-18a since we have also demonstrated that the closed secondary structure is a key determinant to the relative efficient processing of mmu-miR132; however, relaxing the loop with introducing mutations had an adverse effect on miR-132 processing *in vitro*. This implies that the thermodynamic state of the loop of mmu-miR-132 is more important for recognizing protein factors rather than influencing directly the cleavage efficiency. Using the mmu-miR-132 loop in affinity purification we have captured a proteome that partially overlaps with the proteome co-purified with the Microprocessor and it includes DEAD-box, DEAH-box helicases, hnRNP proteins and all members of the TET (FUS, EWS, TAF15) protein family^3^. Among these proteins FUS has already been implicated in facilitating the human miR-132 processing *in vivo* and *in vitro* by promoting Drosha association to the pri-miRNA^48^. In the case of the mouse miR-132 we have found that FUS only elevates miR-132 and miR-212 level *in vitro* if its related protein EWS is inhibited. One explanation for this discrepancy could be the fundamentally different transcriptional organization of miR-132 and miR-212 in mice and humans. In humans, miR-132 and miR-212 are transcribed independently while in mice these miRNAs share the same pri-miRNA and this dissimilarity may require different sets of auxiliary factors for efficient processing. Another possibility is that FUS is required for miR-132 processing in neuronal cells where both the protein and the miRNA have well described cell type specific functions.

Our aim was to identify protein factors that facilitate mmu-miR-132 processing over its co-transcribed and related miRNA, mmu-miR-212. Among the proteins that were shown to specifically bind to the miR-132 loop sequence and were tested *in vitro* for miRNA processing, only p72/DDX17 showed a clear ability to favor miR-132 processing over miR-212, in spite of the fact that knock down of p72 resulted in a significant drop of the recombinant pri-miRNA as well as both mature miRNA levels. This suggests that p72 is responsible for the stability of the primary transcript and additionally could influence the efficiency of mmu-miR-132 processing *in vitro*. p72 belongs to the DEAD box family of RNA helicases and it is implicated in a wide range of processes that regulate endogenous transcription, RNA stability and even viral replication^49^,^50^. p72 has already been described as a cofactor in miRNA processing^10^,^51^. It promotes the maturation of a subset of human miRNAs via its association with the Microprocessor complex; however, until now, miR-132 was not known as a p72 targeted miRNA.

## Material and Methods

### Oligonucletides, siRNAs, PCR primers used in this study

Oligonucleotides for Northern blotting were purchased from GE Dharmacon. mmu-miR-132: UAACAGUCUACAGCCAUGGUCG; mmu-miR-132 complementary sequence: CGACCAUGGCUGUAGACUGUUA (used as a probe for Northern hybridization); mmu-miR-212: UAACAGUCUCCAGUCACGGCC; mmu-miR-212 complementary sequence: GGCCGUGACUGGAGACUGUUA (used as a probe for Northern hybridization); tRNA-Ile complementary sequence: UGGUGGCCCGUACGGGGAUCGA (used as a probe for Northern hybridization).

5′-Biotinylated-2′-*O*-methyl oligos were purchased from GE Dharmacon. mir-132-loop sense 5′-Biotinylated-*2*′*-O-*methyl oligo: CUGUGGGAACCGGAGGUA; mir-132-loop antisense 5′-Biotinylated-2′-*O*-methyl oligo: UACCUCCGGUUCCCACAG; CCC mutant of mir-132 loop sense 5′-Biotinylated-2′-*O*-methyl oligo: CUGUCCCAACCGGAGGUA; *let-7* antisense 5′-Biotinylated-2′-*O*-methyl oligo: UCUUCACUAUACAACCUACUACCUCAACCUU.

siRNA-s were purchased from GE Dharmacon. si-FUS: ON-TARGETplus SMARTpool human FUS siRNA (L-009497-00-0005); si-EWS: ON-TARGETplus SMARTpool human EWSR1 siRNA (L-005119-02-0005); si-hnRNPM: ON-TARGETplus SMARTpool human HNRNPM siRNA (L-013452-00-0005); si-HNRPU: ON-TARGETplus SMARTpool human HNRNPU (L-013501-00-0005); si-DHX36: ON-TARGETplus SMARTpool human DHX36 (L-013167-01-0005); si-HNRPH1: ON-TARGETplus SMARTpool human HNRNPH1 (L-012107-00-0005); si-HNRPH2: ON-TARGETplus SMARTpool human HNRNPH2 (L-013245-02-0005); si-HNRPF: ON-TARGETplus SMARTpool human HNRNPF (L-013449-01-0005); si-KHSRP: ON-TARGETplus SMARTpool KHSRP (L-009490-00-0005); si-p68: CUCUAAUGUGGAGUGCGAC; si-p72: CAAGGGUACCGCCUAUACC.

miRNA PCR primers were purchased from ABI/Life Technologies. hsa-miR-132; mature miRNA Sequence: UAACAGUCUACAGCCAUGGUCG; (ABI/Life Technologies; 4427975); hsa-miR-212; mature miRNA Sequence: UAACAGUCUCCAGUCACGGCC; (ABI/Life Technologies; 4427975); hsa-miR-132-loop; custom-made target sequence: CUGUGGGAACCGGAGGUA; (ABI/Life Technologies; custom-made); hsa-miR-16; mature miRNA Sequence: UAGCAGCACGUAAAUAUUGGCG; (ABI/Life Technologies; 4427975).

### Plasmid DNAs

All the plasmids which were used in this study were generated by the College of Life Sciences Cloning Service, University of Dundee.

The wild type pri-miR-212/132::GFP was generated using the mouse miR132/212 exon-intron-exon (1.46 kb) sequence which was amplified from a mouse BAC (bacterial artificial chromosome) clone (RP23-142A14), and the purified PCR product was cloned into HindIII/BamHI cloning site of pEGFP-N1 (Clontech) in a ligation reaction using HindIII/BglII sites. The resulting full-length clone was fully sequenced.

The mutant pri-miR-212/132::GFP constructs were generated by PCR mutagenesis using KOD Hot Start DNA polymerase (Novagen). The wild type pri-miR-212/132::GFP plasmid was used as a parental plasmid. In the 1483 plasmid the 1158–1160 GGG of the pri-miR-212/132 was mutagenized to CCC (TGTGGGAACCGGAGGT/TGT**ccc**AACCGGAGGT). The following sites were mutagenized similarly in other constructs: 6332 plasmid (TGTGGGAACCGGAGGT/TGTGGGAA**gg**GGAGGT); 1971 plasmid (TGTGGGAACCGGAGGT/TGT**aac**AA**tt**GGAGGT); 1967 plasmid (TGTGGGAACCGGAGGT/TGTG**cc**AA**g**CGGAGGT).

The luciferase reporter plasmid with multiple sites complementary to the miR-132 loop sequence was prepared using the parental vector psiCHECK2 (Promega) containing hRluc and hluc+. The annealed DNA sequence is: tcgaTTGTTACCTCCGGTTCCCACAGTAATTGTTACCTCCGGTTCCCACAGTAATTGTTACCTCCGGTTCCCACAGTAA.

### Q-PCR (quantitative PCR)

Q-PCR for mature miRNA was carried out using TaqMan MicroRNA assays from Applied Biosystems/Life Technologies, according to the manufacturer’s protocols. For relative quantification the fold change was determined relative to the un-treated/un-transfected control, using miR-16 levels to correct for loading. For absolute quantification a calibration curve was made using synthetic miR-132, miR-212 and miR-132-loop sequences (this was generated via a custom assay design pipeline), and based on the calibration curve the absolute amount of the miRNAs in the samples were calculated.

### Antibodies, Western blotting

Samples were run on 4–12% (w/v) Bis-Tris gradient polyacrylamide gels (Invitrogen/Life Sciences) and transfered on to PVDF membranes (Millipore) using standard protocols. Detection was achieved using HRP (horseradish peroxidase)-conjugated secondary antibodies (Jackson) and chemiluminescent substrate (Thermo Scientific Supersignal West Chemoluminescent substrate or with Millipore Immobilon Western chemonluminescent HRP substrate).

### Antibodies used in this study

Human-Ago2 (11A9)^52^; Mouse-Ago2 (4B9): both provided by Gunter Meister’s laboratory; p68 (PAb204)^53^; p72 (SQQ-K14)^53^; GFP: Roche; 11814460001; Tubulin (DM1A): Sigma; T 6199; Drosha: Abcam; ab135956; FUS: Bethyl Laboratories; A300-293A; EWS: Bethyl Laboratories; A300–418A; TAF15: Bethyl Laboratories; A300–307A; DHX36: Abcam; ab70269; hnRNPH1,2 (N-16): Santa Cruz Biotechnology; sc-10042; hnRNPU (C-15): Santa Cruz; sc-13663; hnRNPF (N-15): Santa Cruz; sc-10045; hnRNPM (5-RE36): Santa Cruz; sc-134360; KSRP: Bethyl Laboratories; A302-021A; GAPDH (14C10): Cell Signaling; 2118.

### Cell cultures, transfection

#### Primary cortical neuronal cultures

Primary cortical primary neurons were isolated as described previously^54^, and were cultured in Neurobasal A medium (Invitrogen/Life Tecnologies) supplemented with 2% (v/v) B27 (Invitrogen/Life Tecnologies), 1 mM L-glutamine (Invitrogen/Life Tecnologies), 100 units/ml penicillin and 0.1 mg/ml streptomycin (Invitrogen/Life Tecnologies) and plated on to poly-D-lysine-coated plates (100 μg/ml). The cultures were grown at 37 °C in a sterile incubator in 5% CO_2_.

Cortical neurons were stimulated with 50 ng/ml Brain Derived Neurotrophic Factor (BNDF) (Peprotech) for the indicated time periods, and collected in Trizol for RNA purification. RNA purification was carried out using microRNeasy mini kits (Qiagen) in line with the manufacturer’s protocol.

#### Hela and Neuro2A cells

HeLa and Neuro2A cells were grown in Dulbecco’s modified Eagle’s medium (DMEM) (Invitrogen) supplemented with 10% (v/v) FBS (fetal bovine serum) (Invitrogen), 5 mM L-glutamine (Invitrogen). The cultures were grown at 37 °C in a sterile incubator in 5% CO_2_.

For measuring the turn-over rate of the miRNAs, Hela cells were transfected with wild type pri-miR-212/132::GFP reporter construct for 16 hours, then cells were treated with 1.0 ug/ml Actinomycin-D (Sigma) for the indicated time periods, and harvested in 2 × SDS protein sample buffer for protein detection or in Trizol for RNA purification.

#### Transfections

Hela and Neuro2A cells were transfected with the GFP-reporter plasmids (either wt or mutant 212/132::GFP) for 16 hours using Effectene transfection reagent (Qiagen; 301427), according to the manufacturer’s protocol. The cells were collected in 2 × SDS protein sample buffer for protein detection or in Trizol for RNA purification.

For dual luciferase assays, Hela cells were co-transfected with luciferase-reporter constructs, GFP-reporter constructs and specific miRNA inhibitor for 16 hours using Lipofectamine^®^ 2000 transfection reagent (Invitrogen/Life Technologies) following the manufacturer instructions. The cells were directly lysed in 1× passive lysis buffer (Promega).

For siRNA knockdown experiments, siRNA transfection was done using 20 nM siRNA (Dharmacon) and Lipofectamine^®^ RNAiMAX Transfection Reagent (Life Tecnologies) following the manufacturer protocol. 48 hours after transfection the cells were either directly harvested in 2 × SDS protein sample buffer for protein detection or in Trizol for RNA purification.

### Affinity purification of miR-132 loop associated complexes using biotinylated 2’-*O*-Me-oligos

Cells were plated onto 10 cm dishes, washed with PBS, harvested in 2× mammalian lysis buffer (100 mM Tris-Cl pH 7.4; 0.3 M NaCl; 0.54 M sucrose; 0.2% NP-40; 0.2% Triton-X; 0.2% 2-mercaptoethanol; EDTA-free mini protease inhibitor). After homogenization (dounce homogenizer) and 30 minutes lysis the cell extracts were collected by centrifugation at 13000 rpm for 30 minutes.

Pre-clearing of the lysate and 2′-*O*-Me-oligo pull-down was done in 1× mammalian lysis buffer (50 mM Tris-Cl pH 7.4; 0.15 M NaCl; 0.27 M sucrose; 1% Triton-X; 0.1% 2-mercaptoethanol; EDTA-free mini protease inhibitor).

Binding of the 2′-*O*-Me-oligos (mir-132 as; miR-132 loop CCC; miR-132 loop) to Dynabeads^®^ M-270 Streptavidin (Invitrogen/Life Technologies) was done in 2× Binding and Washing buffer (10 mM Tris-Cl pH 7.5; 1 mM EDTA; 2 M NaCl) for 30 minutes, then equilibrated with 1× mammalian lysis buffer. The 2′-*O*-Me-oligo pull-down was done in 1× mammalian lysis buffer for 16 hours at 4 °C. Then the beads were washed with 1× mammalian lysis buffer 4-times, and re-suspended directly in 50 μl 2× protein sample buffer (125 mM Tris-Cl pH 6.8; 20% glycerol; 4% SDS; 0.04% Bromphenol Blue).

5 ul of protein sample was used for the Ago2 and other protein detection. 45 μl of protein sample was used for 2D PAGE and Coomassie Blue staining (SimplyBlue™ SafeStain; Invitrogen/Life Technoogies) (45 μl was splitted into two and loaded into 2 lanes next to each other). Protein bands labeled with numbers were excised and subjected to mass spectrometry.

### Immunoprecipitation experiments

#### Immunoprecipitations with p68 and p72

Cells were plated onto 10 cm dishes, transfected with wild type pri-miR-212/312::GFP reporter or 2 mutant pri-miR-212/312::GFP constructs for 16 hours using 2 μg plasmid/dish and following the standard Effectene (Qiagen) transfection protocol.

For p72/p68 immunoprecipitation cells were washed with PBS, harvested in 2× mammalian lysis buffer (100 mM Tris-Cl pH 7.4; 0.3 M NaCl; 0.54 M sucrose; 0.2% NP-40; 0.2% Triton-X; 0.2% 2-mercaptoethanol; EDTA-free mini protease inhibitor). After homogenization (dounce homogenizer) and 30 minutes lysis the cell extracts were collected by centrifugation at 13000 rpm for 30 minutes.

Pre-clearing of the lysate and p72/p68 immunoprecipitation was done in 1× mammalian lysis buffer (50 mM Tris-Cl pH 7.4; 0.15 M NaCl; 0.27 M sucrose; 1% Triton-X; 0.1% 2-mercaptoethanol; EDTA-free mini protease inhibitor). After binding the p72 (SQQK14) or p68 (pAB204) antibody to the Dynabeads Protein-G (Invitrogen/Life Technologies) for 30 minutes, the immunoprecipitation was carried out for 16 hours at 4 °C. The beads were washed with 0.5 M NaCl containing 1× mammalian lysis buffer 3 times, then with 0.15 M NaCl containing 1× mammalian lysis buffer. The beads were re-suspended in 50 μl 1× mammalian lysis buffer. 5 μl beads were used for protein detection and 45 μl for RNA purification.

#### Immunoprecipitation with Ago2

Cells were plated onto 10 cm dishes, transfected with wild type pri-miR-212/312::GFP reporter construct for 16 hours using 2 μg plasmid/dish and following the standard Effectene (Qiagen) transfection protocol.

For Ago2 Immunoprecipitation cells were washed with PBS, harvested in NP-40 lysis buffer (50 mM Tris-Cl pH 7.5; 150 mM NaCl; 0.1% NP-40; EDTA-free mini protease inhibitor). After 60 minutes lysis the cell extracts were collected by centrifugation at 13000 rpm for 30 minutes.

Pre-clearing of the lysate and Ago2 Ip was done in NP-40 lysis buffer (50 mM Tris-Cl pH 7.5; 0.15 M NaCl; 0.1% NP-40; EDTA-free mini protease inhibitor). After binding the Ago2 (11A9/Meister) antibody to the Dynabeads Protein-G (Invitrogen/Life Technologies) for 30 minutes, the immunoprecipitation was carried out for 16 hours at 4 °C. The beads were washed with 150 mM NaCl containing NP-40 lysis buffer, then with 300 mM NaCl containing NP-40 lysis buffer, finally with 500 mM NaCl containing NP-40 lysis buffer. The beads were re-suspended in 100 ul 150 mM NaCl containing NP-40 lysis buffer, 15 ul beads were used for protein detection and 85 μl for RNA purification.

### Dual Luciferase assays

Cells were lysed in 1× passive lysis buffer (Promega). Luciferase activity was measured using a dual luciferase reporter assay system (Promega) with a Microlumat Plus LB96V microplate luminometer (EG&G Berthold, Natick, MA).

### Northern Blotting

RNA purification was carried out using microRNeasy mini kits (Qiagen) in line with the manufacturer’s protocol. Detection of small RNAs was performed using sensitive Northern blot method described by Pall *et al.* 2007. In brief, 10 μg of total RNA was separated on 15% (w/v) polyacrylamide gels containing 7 M urea and 20 mM MOPS/NaCl (pH 7.0). After transferring the RNAs by semi-dry blotting on to a Hybond N nylon membrane (Amersham Biosciences), the RNA was chemically cross-linked to the membrane. Detection and quantification of the signals were carried out as described with a slight modification being that the membranes were washed for twice for 1 h at 65 °C with 0.1% SDS and 20× SSC.

## Additional Information

**How to cite this article**: Remenyi, J. *et al.* The loop structure and the RNA helicase p72/DDX17 influence the processing efficiency of the mice miR-132. *Sci. Rep.* **6**, 22848; doi: 10.1038/srep22848 (2016).

## Supplementary Material

Supplementary Information
