# Supplementary material for: The loop structure and the RNA helicase p72/DDX17 influence the processing efficiency of the mice miR-132
Source: Sci Rep. 2016 Mar 7;6:22848. doi: 10.1038/srep22848 (PMC4780006; doi:10.1038/srep22848)

The loop structure and the RNA helicase p72/DDX17 influence the processing efficiency of the mice miR-132.

Judit Remenyi[1], Sarah Bajan[3],Frances V. Fuller-Pace[1,4], J. Simon C. Arthur[2,4], Gyorgy Hutvagner[3,4]

1: Division of Cancer Research, Jacqui Wood Cancer Centre, University of Dundee, Ninewells Hospital and Medical School, Dundee, UK

2: Division of Cell Signaling and Immunology, College of Life Sciences, Wellcome Trust Building, University of Dundee, Dundee, UK

3: Faculty of Engineering and Information Technology, Centre for Health Technologies, University of Technology Sydney, NSW 2007

4: Correspondence to be sent: gyorgy.hutvagner@uts.edu.au, j.s.c.arthur@ dundee.ac.uk, f.v.fullerpace@dundee.ac.uk>

Figure legends for Supplementary Figures

Supplementary Figure 1. (A) Western blotting showing that the reported plasmid (pri-miR-212/132::GFP) designed to study miR-132 and miR-212 processing in vitro expresses GFP. Northern hybridization with miR-132 and miR-212 probes show that this construct also produces bone fide miRNAs in mice (Neuro2A) and human (HeLa) cells. RNA probe detecting tRNA-Ile was used as loading control. (B) The pri-miRNA/miRNA ratios of miR-132 and miR-212 are similar. The same Northern hybridization that was shown in (A) (left panel, HeLa cells) but included the bands those show the oligo hybridization to the respective pre-miRNAs. The relative miRNA/pre-miRNA levels were calculated and presented below each panel (C) Western blot showing the efficiency of PolII inhibition described in Figure 1D. Hela cells were transfected with GFP reported plasmid (Figure 1B) and PolII transcription was inhibited up to 8 hours by administering 1.0 μg/ml Actinomycin D. Western blotting was carried out with GFP and tubulin antibodies.

Supplementary Figure 2. The loop structure affects the ratio of miR-132/miR-212. Repetition of experiment presented in Figure 3 but using Northern blotting for miRNA detection. (A) HeLa cells were transfected with pri-miRNA-212/132::GFP fusion reporters (Figure 3A) and miR-132 and miR-212 were detected with Northern hybridization. Hybridization to $tRNA^{Ile}$ was used as a loading control. (B) Quantification of (A) showing the relative miR-132/212 ratios. Labels of the reporter plasmids are identical to Figure 3A.

Supplementary Figure 3. Testing the effects of the 2′-*O*-Methyl oligos used for affinity purification on the processing of the pri-miRNA-123/212::GFP (Figure 1B) reporter plasmid. (A) Co-transfection of miR-132 loop, miRNA-132 loop CCC and miR-212 loop 2′-*O*-Methyl oligonucleotides with pri-miRNA-123/212::GFP (Figure 1B) Western blotting for GFP was used to visualize the effects of the oligos on pri-miRNA processing. Tubilin was used as a loading control. (B) In the experiment described in (A) miRNA-132 and miR-212 levels were quantified using Taqman qPCR. M means transfection with only transfection reagent.

Supplementary Figure 4. Affinity purification of mmu-miR-132 loop associated proteins. (A) Coomassie Blue staining of the 2D PAGE gel of the proteomes affinity purified with biotinylted 2′-O-methyl oligos. mir-132 loop: biotinylated 2′-*O*-Methyl oligo mimicking the sequence derived from the miR-132 loop. miR-132 AS: biotinylated 2′-*O*-Methyl oligo complementary to miR-132 loop. miR-132 loop CCC: biotinylated 2′-*O*-Methyl oligo similar to miR-132 loop only three GGG sequence was replaced with CCC (see mutation in Figure 3B clone: 1483). Protein bands labeled with numbers were excised and subjected to mass spectrometry. (B) List of the proteins identified in the excised gel slices. Only proteins that are identified with three or more peptides are enlisted. Numbers in brackets indicate the number of peptides were identified from the corresponding protein.

Supplementary Figure 5. Testing the effect of the miR-132 loop associated proteins on the processing of pri-miRNA 212/132 and the accumulation of miR-132 and miR-212 in vitro. (A and B) pri-miR-212/132::GFP reporter was co-transfected with siRNAs against proteins specifically binds to mmu-miR-132 loop into HeLa cells and the level of miR-132 and miR-212 was quantified using PCR. The ratios of mature miR-132/212 are indicated in the graph (left panel). The efficiency of the siRNA knockdowns was monitored with Western blotting and GFP antibody was used to measure the level of pri-miR-212/132::GFP (right panel). C and control: transfection carried out with non-targeting siRNA.

Supplementary Figure 1

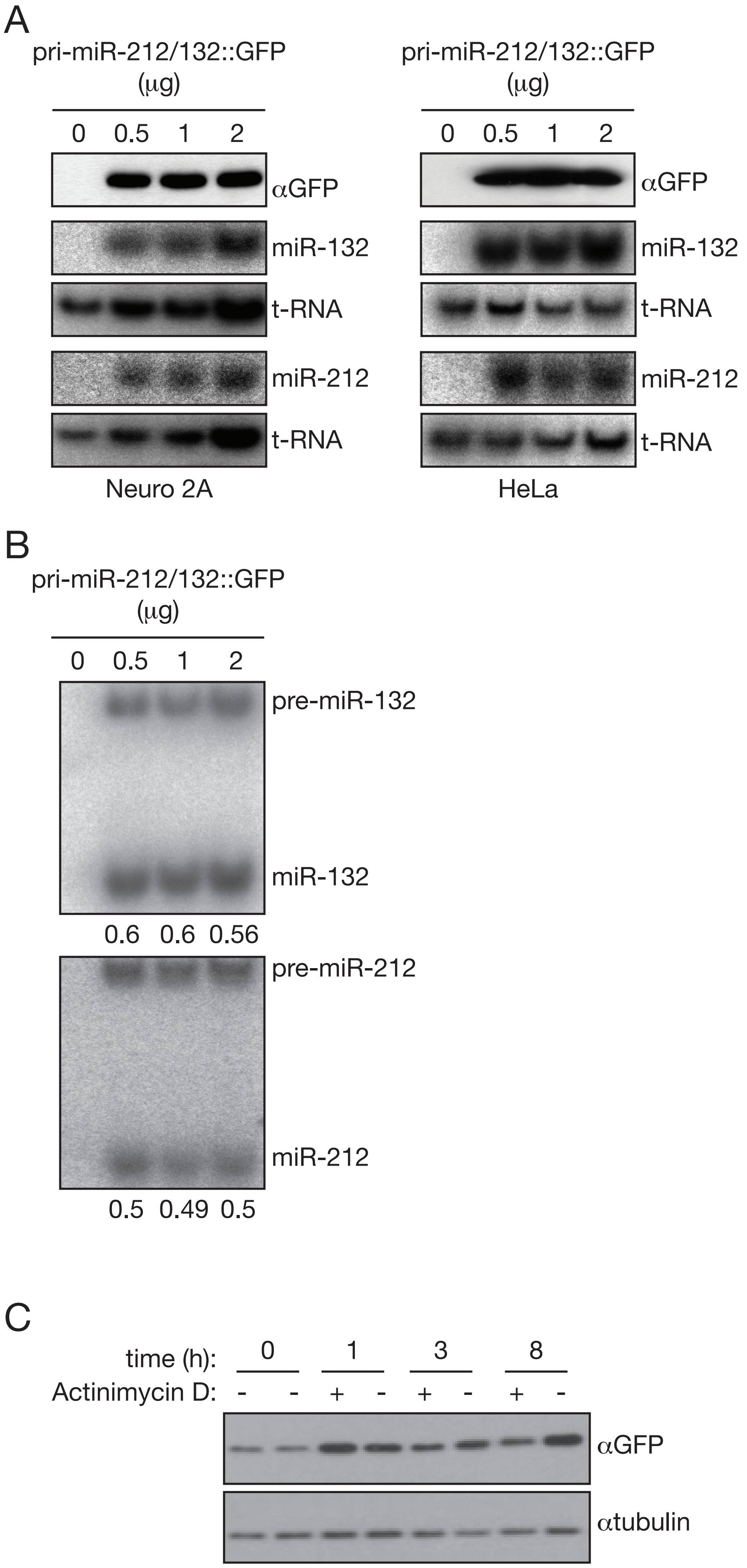

Supplementary Figure 2

A

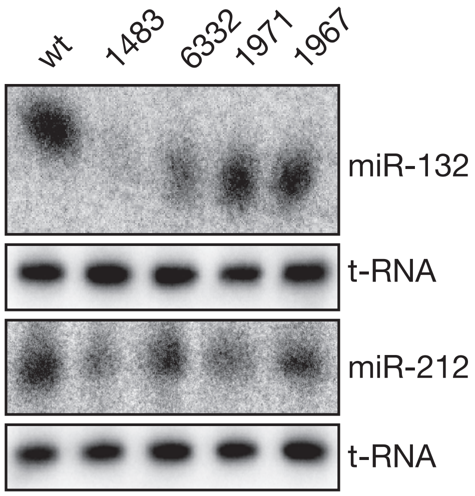

B

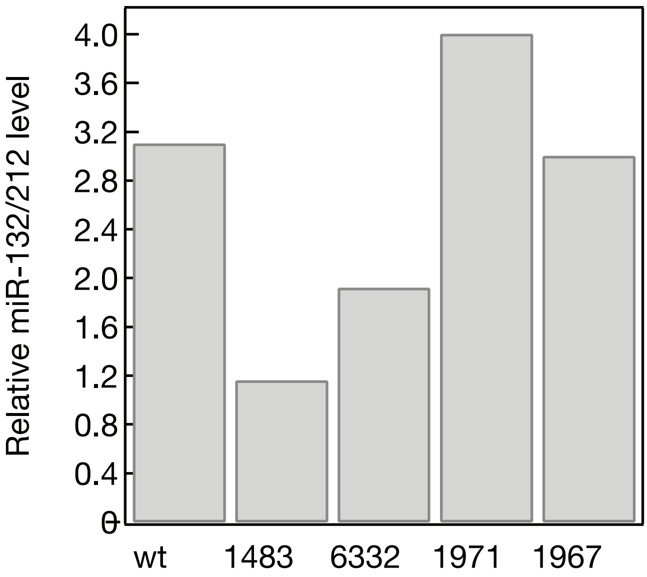

Supplemantary Figure 3.

A

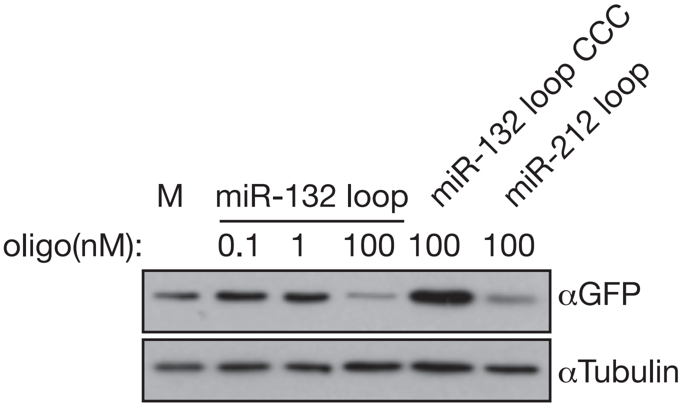

B

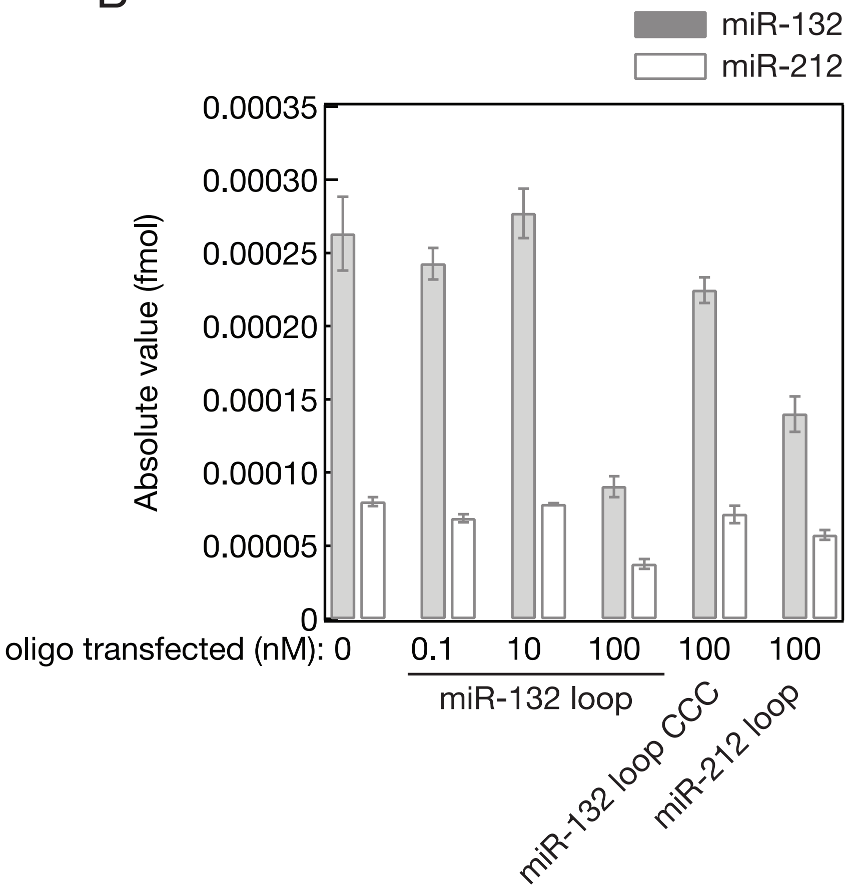

A

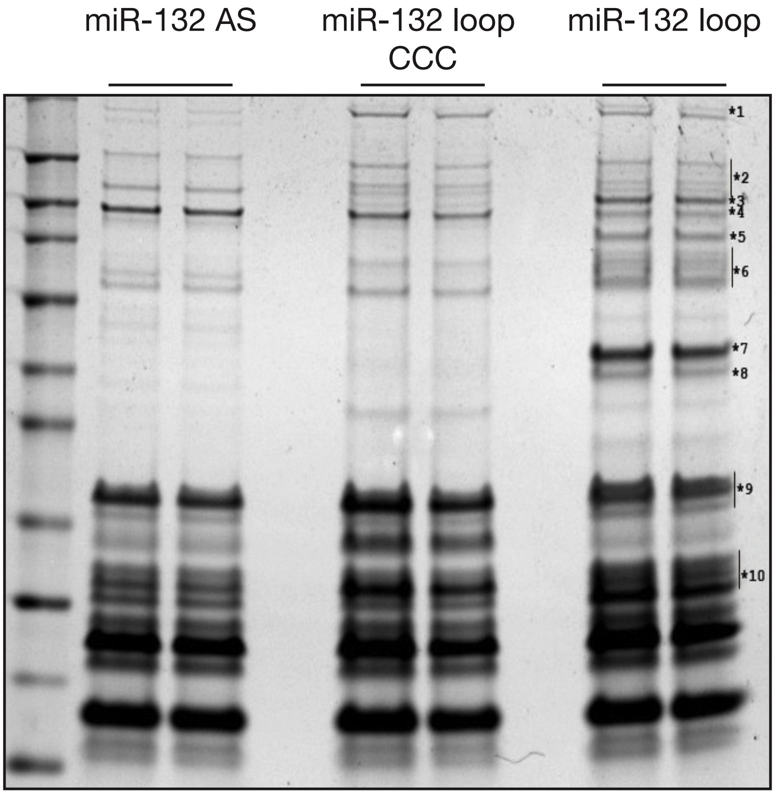

B

number	proteins identified
1	NUMA1(6)
2	SKIV2L2(10), MYBBP1A(12), SLTM(3), MAP7D3(5), RRP12(8), DHX36(3)
3	DHX36(27), HNRNPU(4)
4	AP2A1(7), AP2B1(6), ASPH(3)
5	EWSR1(9)
6	HNRNPM(59), GNL3(30), RMB14(16), FUS(10), EWSR1(4), TAF15(4), DDX5(4)
7	HNRNPH1(74), HNRNPH2(38), HNRNPF(15), TUBA1C(5), GRSF1(3), AP2M1(3)
8	HNRNPF(34), HNRNPH1(17), RBMX(4)
9	RPS6, RPS4X, RPS2, HIST1H1C, H1FX, SLC25A5,
10	RPL24, RPL23A, RPS24, RPL26L1, RPS11, RPL11, CNBP

Supplementary Figure 5

A

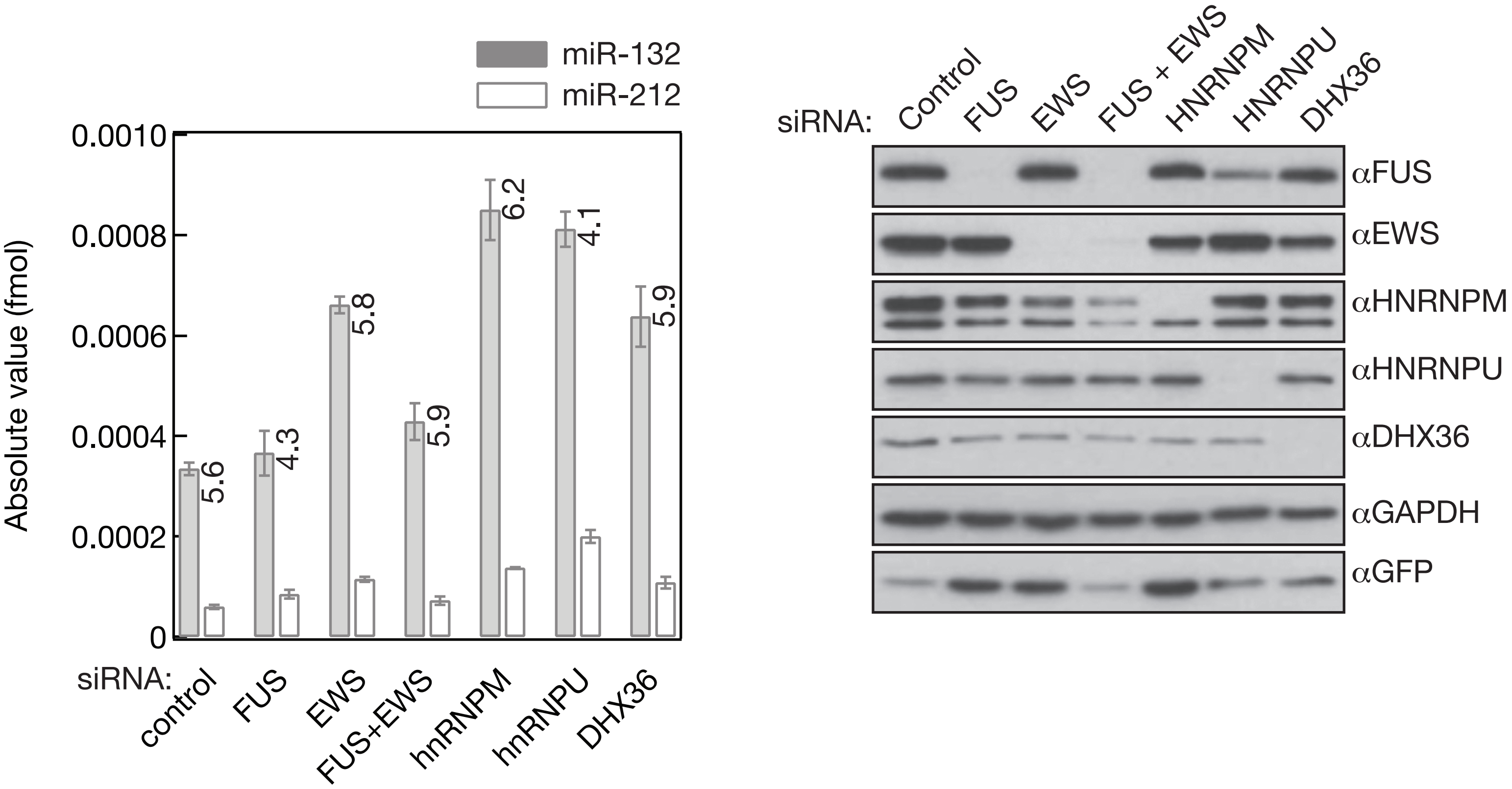

B

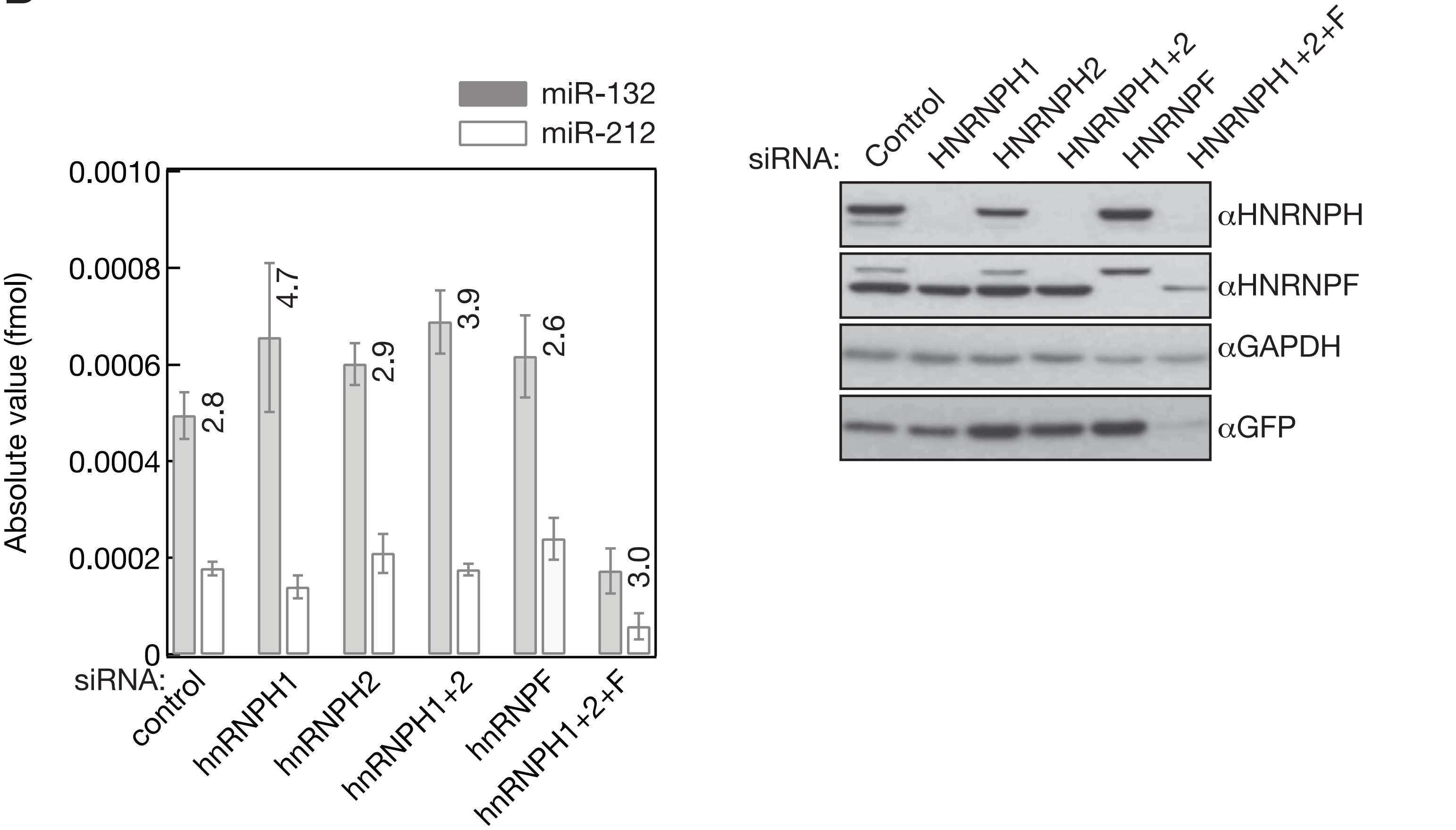

Supplement: Supplementary Information [file srep22848-s1.pdf]
